# Operating Room Protocols and Infection Control

**DOI:** 10.1007/978-981-15-1346-6_9

**Published:** 2020-06-24

**Authors:** Rishi Kumar Bali

**Affiliations:** 1 Bhagwan Mahaveer Jain hospital, Bangalore, India; 2 Associate Professor, SRM Dental College, Ramapuram, Chennai, Tamil Nadu India; 3 Ananthapuri Hospitals & Research Institute, Kerala Institute of Medical Sciences, Trivandrum, Kerala India; 4 Department of Maxillofacial Plastic Surgery, Uppsala University Hospital, Uppsala, Sweden; 5 Associate Professor, Department of Dentistry, All India Institute of Medical Sciences, Bhopal, Madhya Pradesh India; Postgraduate Department of OMFS, DAV Dental college hospital, Yamunanagar, Haryana India

**Keywords:** Sterilization and Disinfection, Operating Room, Infection Control, Surgical Safety

## Abstract

In the modern day’s Oral and Maxillofacial surgical practice, complex surgical and aesthetical procedures are being carried out associated with an increased risk of infectious complications. Therefore, to ensure better outcomes of the surgical procedures, it is absolutely necessary that appropriate measures must be taken to decrease the incidence of associated infections. The practices to be carried out for infection control include proper scrubbing procedures for both patient and the operator, specific protocols to be followed by the operating personnel at the time of procedures, proper handling of the instruments and maintaining an aseptic environment throughout the procedure. The main aim of this chapter is to provide information on the preoperative, operative and post-operative protocols that should be adhered to improve the safety of the patients undergoing surgical procedures.

## Introduction

A study comprising data from 56 countries in 2004 stated that the annual major surgery volume was estimated to be 187–281 million operations, accounting for approximately one operation annually for every 25 human beings alive [1]. In subsequent studies, data were obtained from a total of 194 Member States of the World Health Organization for the years 2005–2012. According to these studies, 312.9 million operations took place in 2012, showing an increase from the 2004 estimate of 226.4 million operations. 6.3% and 23.1% of operations were carried out in *very-low* and *low*-expenditure countries representing only 36.8% (2573 million people) and 34.2% (2393 million people) of the global population of 7001 million people, respectively [2]. The incidence of postoperative infections reported among the developed countries like UK and USA was approximately 5% and 5–6%, whereas in developing countries like India it is much higher, accounting for approximately 10–25%. [3, 4].

The main problem encountered in the practice of surgical safety is that existing safety practices are not adequate in some countries. Lack of resources is the main reason behind this, particularly in developing countries. Good infection prevention and control is essential to ensure the safety of the patient undergoing any surgical procedure in the operating theater. The surgical site infections (SSIs) constitute 20% of the total hospital-acquired infections [4]. These infections cause substantial patient mortality and morbidity and burden healthcare systems with massive costs. Since these infections are primarily acquired during the operative procedure when the wound is still open, stringent protocols need to be followed at this point to minimize their onset.

## Terminology

To establish surgical protocols, it is important to understand the basic concepts of sterilization, asepsis, and infection control. In this respect, the following terminologies are very commonly used:

### Antibiotics

These agents are a by-product of certain microorganisms, which either have the capacity to destroy or inhibit the growth of other microorganisms at low concentrations.

### Anti-Infective

A substance (or drug) capable of killing microorganisms or inhibiting their growth, in particular, pathogenic microorganisms. This is a general term used to encompass those drugs that specifically act on certain types of microorganisms, including antibacterial (antibiotics), antifungal, antiviral, and antiprotozoal agents.

### Antimicrobial Agent

Any agent synthetically or naturally obtained that can destroy or attenuate the microorganisms.

### Antisepsis

It is the process in which microbial agents on a living surface are either killed or their growth is arrested.

### Antiseptic

These are the substances applied on the living tissues to reduce the possibility of infection, sepsis, and putrefaction by inhibiting the activity or growth of the microorganisms.

### Asepsis

The state of being free from living pathogenic organisms.

### Aseptic

Free of or using methods to keep free from microorganisms.

### Aseptic Processing

It is defined as the processing and packaging of a sterile product into sterilized containers followed by proper sealing with a sterilized closure in a manner to control microbiological recontamination.

### Bactericide

It is an antimicrobial agent that has the capacity to destroy both nonpathogenic and pathogenic organisms but may not destroy bacteria in spore form.

### Bacteriostatic

It is an antimicrobial agent that inhibits the growth of microorganisms but is not capable of killing them.

### Bioburden

The occurrence of viable microorganisms on a surface or object before the sterilization procedure.

### Biologic Indicator (BI)

A standardized test preparation of bacterial spores used to demonstrate effective sterilizing conditions by providing a defined resistance to a specific sterilization process.

### Chemical Indicator

These are agents or devices used to monitor or confirm the attainment of one or more of the parameters required for a satisfactory sterilization process or used in a specific test of the sterilization equipment.

### Chemisterilant

It is an agent, chemical in nature with properties that kills all forms of microbial agents, including spores.

### Cleaning

It is the process of removing all forms of foreign materials (from objects using detergents & water, soaps, and enzymes) by employing the mechanical action of washing or scrubbing the object.

### Contamination

It is the process of entry of microbial agents into tissues or any aseptic environment.

### Crossinfection

The spread of infection from one person, object or place to another.

### Decontamination

The process by which a person or a surface is made free from all the agents that contaminate the surface and lead to the spread of infections. [5]

### Detergent

It is a chemical agent with cleansing actions in dilute solutions, which, on combining with impurities and dirt, make them more soluble.

### Disease

Disruption of the normal performance of the vital functions of a plant or animal by an infection.

### Disinfectant

This is an agent, usually a chemical, applied on inanimate objects that destroys microorganism in the vegetative form but not the spores.

Chemical disinfectant agents are categorized into low level, intermediate, and high level (depending on the product claims and regulatory requirements in different parts of the world).

- High-level disinfection (HLD): It is a process in which a small number of spores or certain bacteria are killed by the use of certain antimicrobial agents at a specific temperature and appropriate concentration.
- Intermediate-level disinfection (ILD): It is a process in which vegetative forms of all microorganisms are destroyed but affect the activity of spores of certain bacteria.
- Low-level disinfection (LLD): It is a process in which vegetative forms of all microorganisms are destroyed having no activity on spores of bacteria at very low concentrations.

### Disinfection

Antimicrobial process to remove, destroy, or deactivate microorganisms on surfaces or in liquids. Disinfection is often considered as a reduction of the numbers and types of viable microorganisms (or “bioburden”) but may not be assumed to render the surface or liquid free from viable microbial contamination (in contrast to sterilization).

### Droplet Nuclei

These are those particles of 1–10 μm that are implicated in the spread of airborne infections.

### Exogenous Infection

The infecting microorganism comes from an external source.

### Fomites

Any inanimate object that is capable of absorbing or transmitting infectious microorganisms from one person to the other.

### Fumigation

The process of disinfecting or purifying an area or object with the fumes of certain chemical agents.

### Germicide

Agents that are designed to kill and destroy pathogenic organisms on the surface of different things.

### Infection

It is the process of invasion of the tissues by microorganisms and their multiplication in the body of the host to produce disease.

### Microorganisms or Microbe

Microscopic organisms, which may exist in its single-celled form or in a colony of cells.

### Minimum Effective Concentration (MEC)

The lowest concentration of a chemical or product, used in a specified process that achieves a claimed activity.

### Minimum Recommended Concentration (MRC)

The lowest concentration of a chemical or product specified by the equipment manufacturer to be used in a process.

### Nosocomial

This comes from two Greek words, i.e., “*nosus”* meaning “*disease*” and “*komeion”* meaning “*to take care of.”* Also known as “hospital-acquired infections.” These are the infections originating or taking place in a hospital.

### Operating Room (OR)

The operating room or operating theater is a facility within a hospital where surgical procedures are carried out in an aseptic environment.

### Pathogen

A pathogen is a tiny living organism, such as a bacterium or virus that is capable of producing disease in an individual.

### Resistance

It is the natural ability of the agent to oppose the effects of any harmful agents.

### Soil

Natural or artificial contamination on a device or surface following its use or simulated use.

### Sterile Barrier System

Packaging that prevents the ingress of microorganisms following a sterilization process, thereby preserving the sterile state.

### Sterilizer

Equipment designed to achieve sterilization.

### Sterilizing Agent

Physical or chemical agent (or combination of agents) that has sufficient microbicidal activity to achieve sterility under defined conditions.

### Septic

Contaminated or infected.

### Spores

These are the reproductive forms of some microorganisms that can survive harsh environmental factors and have the capability of developing into new viable microbes.

### Sterilization

Sterilization is a process that destroys or removes all microbial life completely, including spores by means of certain chemical or physical processes.

### Sterile

Free from living microorganisms.

### Sterilize

Total destruction of all living forms.

### Vector

It is an organism that does not cause disease itself but which spreads infection by conveying pathogens from one host to another.

### Virulence

It is a pathogen’s ability to infect, sustain, or spread infection in a living a host. Historical background of present day protocols is enumerated in Table 9.1.

**Table 9.1:** Historical background leading to proper sterilization and disinfection protocols

Year	Event
• First century BC.	*Varo and Columella* postulated that diseases were caused by invisible beings, “animals minutia,” inhaled or ingested
• 500 AD	*Sushruta* instructed operating team members to clean and fumigate the operating theater with vapors of certain disinfectants prior to all surgical procedures
• 1493–1541	*Paracelsus*, called the father of medicine, reformed pharmacopeia and introduced compositions of lead, copper, sulfur, iron, and mercury
• 1546	*Fracastorious* proposed a “contagion vivum,” as the possible cause of infectious diseases.
• 1827–1912	*Joseph Lister*, “father of modern surgery,” demonstrated that antisepsis could prevent infections; also known as “Listerian era”
• 1889	*William Stewart Halsted* introduced rubber gloves for his scrub nurse
• 1882	*Robert Koch* introduced the use of mercuric bichloride as antiseptic agents and isolated the bacilli of tuberculosis
• 1880s and 1890s.	Sterilization of instruments, hand washing, and the wearing of masks, caps, gloves, and gowns was introduced

## Surgical Site Infections

Approximately 2–5% of all surgical patients tend to acquire surgical site infections (SSIs) [4]. In developed & high-income countries (HICs), SSIs are the second most common cause of healthcare-associated infections [6], whereas in Low- & Middle-Income Countries (LMICs) or underdeveloped & developing countries these infections are the most common ones. Thus, to reduce the risk of surgical site infections, a more systematic approach has to be adopted, based on proper knowledge regarding the status of the patient, type, & time of the operation, personnel involved and the health care facilities available during a surgical procedure. The main pathogenic source of surgical site infections is the endogenous flora (usually aerobic gram positive cocci) of the patient present in the skin, the mucous membranes, or the hollow viscera. The exogenous sources of infection include members of the surgical team, environment of the operating theater and tools, materials & instruments brought to the sterile zones during the surgical procedure. Various strategies employed to prevent or control the occurrence of surgical site infections include reducing the contamination by microorganisms on the sterile surgical instruments as well as the body of the patient, prophylactic preoperative antibiotic coverage, carrying out the surgical procedure carefully, proper handling of the operating room.

## Surgical Safety

Surgical safety is of utmost importance in order to prevent major and life-threatening complications leading to undue loss of life and patient morbidity. Thus, a list of ten essential objectives with a surgical safety checklist have been elucidated by the WHO to be followed by all the personnel present in the operating room to reduce the risk of such complications [7] (Tables 9.2 and 9.3).

**Table 9.2:** WHO: Ten essential objectives for safe surgery

(1) The operating team must ensure that the correct surgical procedure is to be carried out on the correct patient.
(2) The operating personnel should have adequate knowledge regarding the anesthesia, its methods of administration as well as its effects so that minimum pain is experienced by the patient.
(3) The operating personnel should be well prepared for any life-threatening conditions like loss of airway or respiratory function.
(4) The operating team should be prepared for risk of high loss of blood.
(5) The operating team should be well versed with the history of the patient in order to prevent or induce any allergic or adverse drug reactions that can cause a significant risk for the patient.
(6) Care must be taken to minimize the formation of surgical site infections by using proper measures.
(7) Proper care must be taken to not leave any instrument or any foreign material at the surgical site.
(8) All specimens should be carefully identified and secured for further investigations.
(9) Proper communication must be present among the operating team personnel for the safe conduct of the surgical procedure.
(10) A routine surveillance of the surgical volume, safety protocols, capacities, and the outcomes must be carried out by all the hospitals and the health care systems.

**Table 9.3:** WHO’s Surgical Safety Checklist

Before induction of anesthesia	Before skin incision	Before patient leaves operating room
Sign in	Time out	Sign out
Patient has confirmed • Identity • Site • Procedure • Consent	Confirm all team members have introduced themselves by name and role	Nurse verbally confirms with the team • The name of the procedure recorded • The instrument, sponge, and needle counts are correct (or not applicable) • How the specimen is labeled (including patient name) • Whether there are any equipment problems to be addressed
Site marked/not applicable	Surgeon, anesthesia professional and nurse verbally confirm • Patient • Site • Procedure	
Anesthesia safety check completed	Anticipated critical events • Surgeon reviews: What are the critical or unexpected steps, operative duration, anticipated blood loss • Anesthesia team reviews: Are there any patient -specific concerns • Nursing team reviews: Has sterility (including indicator results) been confirmed? Are there equipment issues or any concerns?	Surgeon, anesthesia professional and nurse review the key concerns for recovery and management of this patient
Pulse oximeter on patient and functioning	Has antibiotic prophylaxis been given in the last 60 min? • Yes / not applicable Is essential imaging displayed • Yes/not applicable	
Does patient have a known allergy • No/yes Difficult airway /aspiration risk • No • Yes, and equipment /assistance available Risk of >500 ml blood loss (7 ml/kg in children) • No • Yes and adequate intravenous access and fluids planned		

## Environmental Control and Design

### Operating Room (OR)

The operating room or operating theater is a facility within a hospital where surgical procedures are carried out in an aseptic environment. Since the operating theater is a highly sterile, aseptic, and restricted area in a hospital setting, it is mandatory for all the personnel concerned to have a proper understanding of the working of the operation theater abiding by certain laws, regulations, and professional guidelines. Integrated infection control in the operation theater is the key to decreasing morbidity and mortality among the patients undergoing surgery.

Following essentials must be present in an operating room:

- Adequate lighting
- Proper ventilation with 20 (ACH) air changes/hour
- Correct and sufficient instruments needed in the surgery
- Proper machines and equipment for monitoring the condition of the patients during the surgeries
- Emergency drugs and other items
- Separate rooms should be present to carry different procedures

- Location of the Operating Theater: The location of the operation theater should be such that adequate natural light and proper ventilation is present. For this, a two- or three-story building away from the general hustle and bustle of the hospital is preferred.
- Operation rooms: The standard recommended size of the operation theater is 6.5 m × 6.5 m × 3.5 m and can be modified as per requirement
- Doors and Windows: There should be separate entry and exit doors. The width of the door should be approximately 1.2 to 1.5 m. Sliding doors are preferred than spring-loaded doors in order to minimize the generation of the air currents during the opening and closing of the doors. Windows made from glass are preferred, which are to be planned on one side only.
- The surface/flooring: The flooring must be such that it is nonslippery, strong, & with minimum joints to decrease the accumulation of dust and other tiny particulate matter within it.
- Walls: Washable light and soothing painted walls should be present
- Ceiling: Hard, impervious surface, plaster painted ceiling should be present.

### Different Zones of Operation Theater Complex

**Fig. 9.1:**
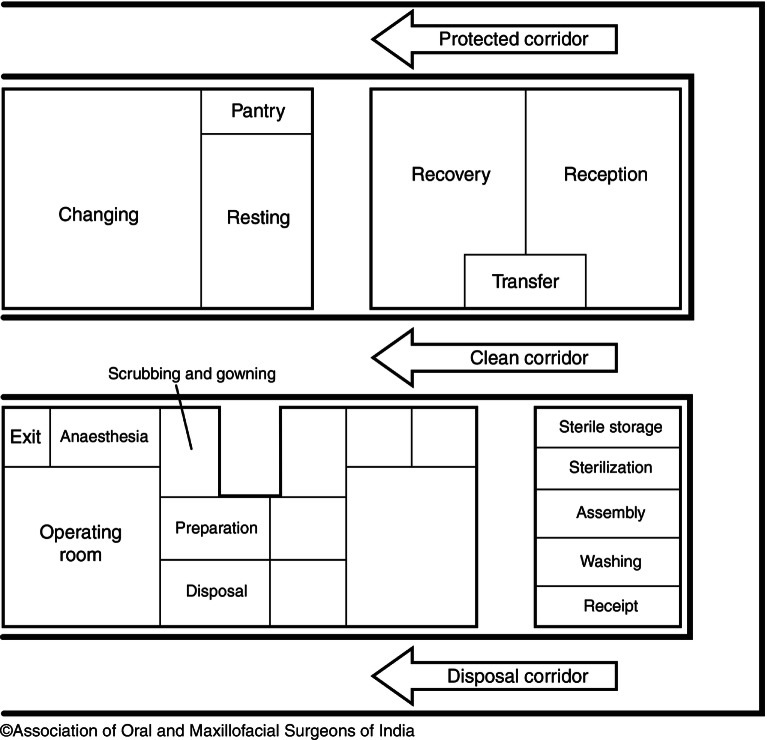
Different zones of operation theater complex

There are four different zones observed in any operating room complex described on the basis of type of cleanliness present, presence or absence of microorganisms, and the different procedures to be carried out in each zone (Fig. 9.1).

1. *Protective zone*: It includes
  Changing room for all the concerned persons
  Transferring passage for the materials, equipment, and the patient
  Rooms for administrative staff
  Storage & record maintenance
  Pre- and postoperative
  Intensive and Coronary Care Units
  Storage rooms to keep the sterilized objects
2. *Clean zone*: Links the protective zone to the aseptic zone
  Clean rooms
  Storage room for equipment
  Rooms designed for surveillance, maintenance, and firefighting
  Exit areas in case of emergencies exists
3. *Aseptic zone:* houses the operating rooms
4. *Disposal zone:* separate exit for disposing contaminated linen /used materials and instruments

### Important Points

- The sterilized materials are transported from a sterile area to the operation theater on a covered cart, thus preventing any accumulation of the dust particles on their surfaces.
- Before entering the operation theater, all supplies/materials must be removed from their shipping or transported containers.
- All the blood-coated or soiled instruments/equipment must be transported in a covered wrap or container from the operating room to the decontamination or reprocessing area.
- Care must be taken that the soiled instruments/equipment must not to be stored with the sterilized goods.

### Maintenance in the Operation Theater

- The machinery must be surveyed at least every week.
- Proper ventilation should be checked regularly and the filters should be changed as required.
- At the time of maintenance or inspection or observation of any fault in the working of the operation theater, the members of The Infection Control team should be informed. The theater should be thoroughly examined by the members of the team and appropriate measures must be taken to maintain the infection control protocols. The operation theater must only be functional after being carefully evaluated and cleared by the infection control team.
- Back-up facility for operation theater in all aspects should be present in any setup to provide any uninterrupted sessions at the time of maintenance or any fault observed.

## Disinfection and Sterilization

Sterilization is the ultimate procedure in controlling the undesired activities of microorganisms that are outside of the human body. Its purpose in the operating field is to prevent the spread of infectious disease, and in surgery, it primarily relates to processing reusable instruments. Surgical instruments are an integral part of surgical field and, being reusable, have greater chances of spreading the microorganisms if any of the reprocessing steps fails. Steps of reprocessing include cleaning, repackaging, disinfection or sterilization, and reusing. Cleaning, being the first step in the cycle of reprocessing, is the major step in the removal of any organic matter present on the surface of the instruments. Any failure in the removal of the visible soil at the initial stage can create a discrepancy in the efficacy of the subsequent disinfection and sterilization procedures. Sterilization is more effective a process than disinfection. The process of disinfection is carried out with the use of various chemical agents. Chemical disinfecting agent necessarily does not kill all microorganisms or spores present on an inanimate object but instead reduces the number of microorganisms to a level that is not harmful to health. Depending on their potency against microbes, they are classified as *High-*, *Intermediate-,* or *Low-level* disinfectants.

The type of the sterilization procedures to be carried out for an object depends upon the classification of the instruments based according to the Spaulding Classification of the medical devices, the type of material of which the object is made of, the microorganisms to be present on the object, and availability of the sterilization methods and equipment (Table 9.4).

**Table 9.4:** Spaulding Classification of Medical Devices And Level of Disinfection

• Critical: An instrument that has a direct contact with sterile tissues or vascular system [8]; such items are to be sterilized and made free from all microorganisms. Examples are extraction forceps, scalpel blades, bone chisels, periodontal scalers, surgical burs, needles
• Semicritical: An instrument that does not usually penetrate the sterile tissues but does come in contact with intact mucous membrane. These items are made free from microorganisms by high-level disinfection. Examples include endoscopes, amalgam condensers, air/water syringe, impression trays, dental hand piece, dental mirrors
• Noncritical: An instrument that does not touch the patient directly or come in contact with the intact skin only. These items may be cleaned or disinfected by low-level disinfection. Examples include light arm/handles, dental chair, dental X-ray equipment, chair side computers, chair switches.

### The Instrument Processing (Decontamination Steps) (Fig. 9.2)

- Various methods of decontamination include.
  - Physical cleaning.
  - Water purification
  - Ultrasonic cleaning
  - Disinfection
  - Antisepsis
  - Sterilization

**Fig. 9.2:**
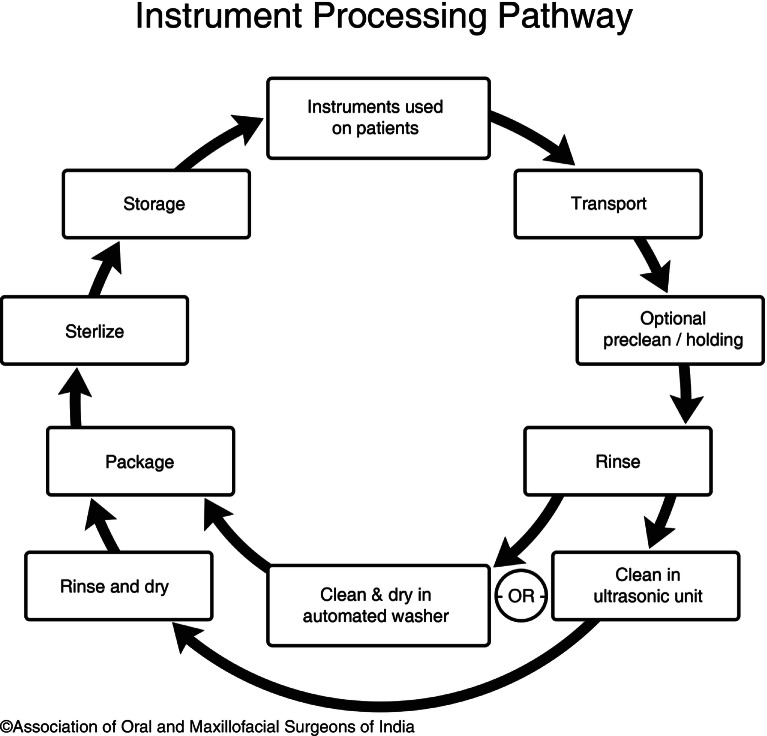
Instrument processing steps

Processing of the clinical or surgical items is a two step procedure.

A. **Cleaning**, being the first step, is the most essential one, which is succeeded either by Disinfection or Sterlization
  Cleaning is the process of removing all the foreign particles present on the surface of the object, which is accomplished by means of two main steps, i.e., cleaning by friction to remove foreign particles and rinsing away by fluids to remove the debris so cleaned.
  If the objects to be sterilized, remain soiled with foreign materials, the microorganisms will be trapped in the organic matter and may interfere with the proper sterilization or disinfection procedure. Therefore, thorough cleaning should always precede the sterilization process.
  Cleaning may be manual or mechanical and is normally accomplished by the use of water, detergents, and mechanical agents. Detergent is essential to dissolve proteins and oil that can reside on instruments and equipment after use.

**Fig. 9.3:**
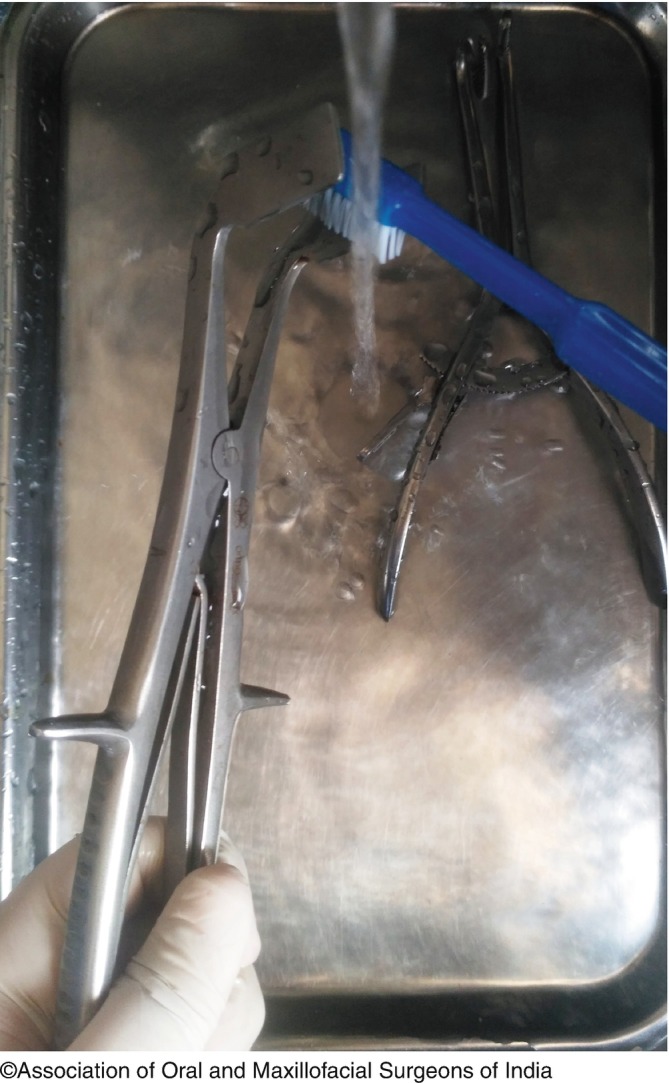
Manual Cleaning

1. *Mechanical cleaning*
  With the advancement in the sterilization equipment, most units are automated and there is very less handling of dirty equipment by the concerned staff. The equipment to be processed is placed in trays and is ready for washing.
  *Washing machine*: It gives a cold rinse followed by a hot wash at 71 °C for 2 min. This is followed by a 10-second hot rinse at 80–90 °C and then by dry heat at 50–75 °C.
  *Ultrasonicator*: The ultrasonicator is a device, which is extremely efficient in removing the debris. 0.44 W/cm^3^ of power is used to remove the debris by the process of sonic waves produced. The solution used most often to clean is an enzymatic presoak (protease formula that dissolves protein).
2. *Manual cleaning*
  It is an active method that is carried out by thoroughly brushing the item with the help of a toothbrush under water to prevent the release of aerosols. The brush should be thoroughly cleaned after use and should be dried. The cleaned items are then dried and made ready for the proper sterilization procedure to be carried out depending upon the material of which they are made and the use they perform.
  Manual cleaning is necessary when:
  Cleaning of instruments by mechanical means is not possible
  Instruments to be cleaned are delicate in nature
  Objects to be cleaned have a narrow lumen (Fig. 9.3)
3. *Soaking of instruments prior to cleaning:*
  There are times when cleaning alone cannot remove the debris present on the surface of the objects as the items become highly soiled with foreign materials. For this, it is sometimes necessary to soak instruments/objects prior to cleaning. A container having a deeper base is filled with detergent & water and all the instruments are kept in it for 3–5 min. The solution prepared is agitated by shaking it vigorously. The cleaned instruments are now removed from the container and placed over a tray for air drying.

### B. Disinfection and Sterlization

**Fig. 9.4:**
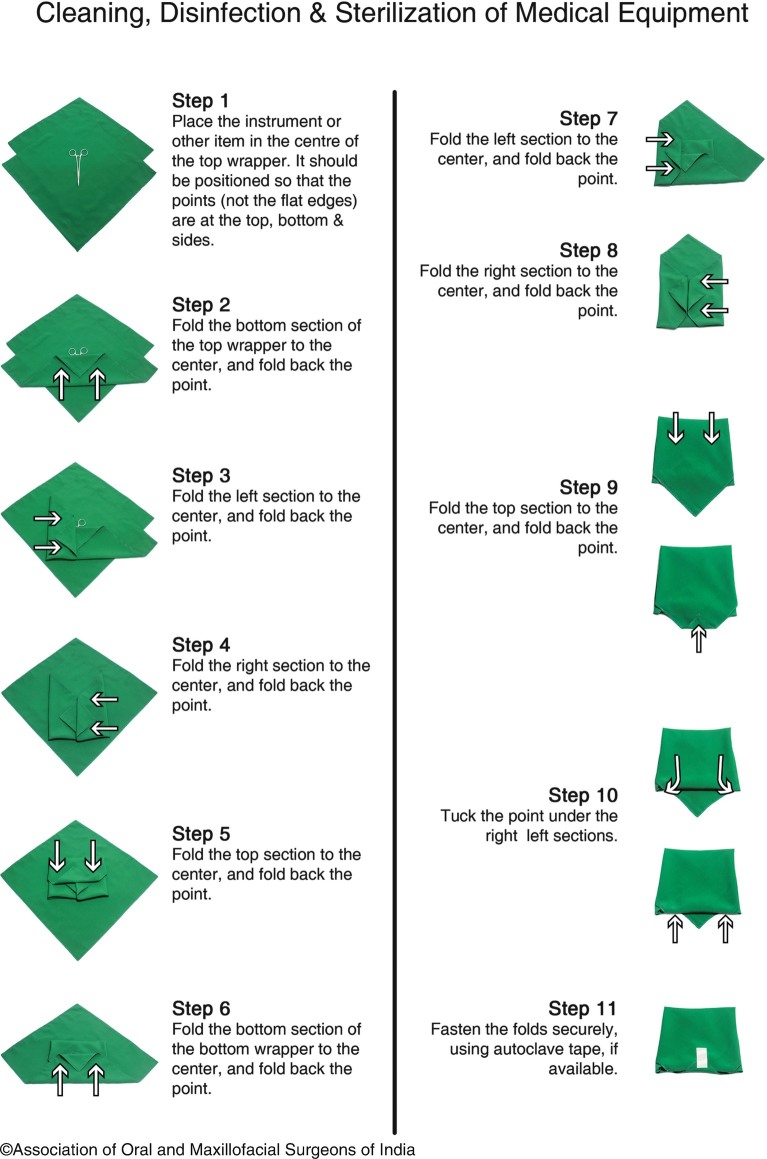
Diagram depicting the way the instruments are wrapped for sterilization

- *Disinfection* can be achieved by either thermal or chemical processes. The thermal disinfection procedure is more easily controlled, more reliable, nontoxic and leaves no residue on the surface and is preferred more than chemical disinfection procedures. The main indication of chemical disinfectant use is the heat-sensitive objects. Chemical High-level disinfection (HLD):

- Most commonly used for heat-labile instruments/objects (e.g., endoscopes) where single use is not cost effective.
- Disinfectants used for this purpose are
  Glutaraldehyde: 2% for 20 min.
  Hydrogen peroxide: 6%–7.5% for 20–30 min.
  Per acetic acid: 0.2–0.35% for 5 min.
  Ortho-phthalaldehyde (OPA) for 5–12 min.
- Steps:
  All items to be disinfected are cleaned and dried.
  Fresh disinfectant solution should be made each day in a sterile container. If a previously prepared solution is to be used, an indicator strip is dipped in the solution to check for the effectiveness of the solution.
  Open all hinged instruments and disassemble whichever possible.
  Place all items in the solution completely submerged in the container.
  The container is covered and the instruments are allowed to soak in for 20 min.
  Remove the items using dry, high-level disinfected pickups.
  Rinse thoroughly with boiled water.
  Air-dry by placing on disinfected tray.
  Items disinfected are covered in a disinfected container and used within a week.

- *Sterilization*
  Sterilization is a method by which an article, medium, or surface is made free from all microbial invasions, including spores. The main aim of sterilization of instruments is proper delivery of sterilized instruments at the operating field, thereby maintaining a sterile environment and reducing the spread of infections from one person to another. Proper handling of the sterilized instruments is done by appropriate wrapping and storage of the instruments, thereby increasing the shelf life of the sterilized instruments. The instruments should be bagged or wrapped in a muslin cloth or clear pouches or paper before and after the procedure and the wrapping should be sealed with tape. No pin, staple, or any paper clips are to be applied on the wrapping as these may create small openings, which may allow entry of microorganisms, thereby hampering the process of sterilization. Sterilization is accomplished by:

1. *Steam sterilization (autoclaving):-*

- This is the most simple and efficient means of sterilizing instruments. It is also commonly called steam sterilizing or autoclaving.
- The steam autoclaves best suited for outpatient practice are usually made to operate in the following range.
  - Temperature 121 °C (250 °F) at a pressure of 15 pounds per square inch (psi) for 15 min.
  - Temperature 134 °C (270 °F) at a pressure of 30 psi for 3 min. This process termed “flash sterilization” has practical use in the operating room where fast sterilization of instruments may be necessary.
- This combination of moisture and heat provides the bacteria-destroying power currently most effective against all forms of microorganisms.
- Mainly used for items that are wrapped or porous.
- Autoclaves are either classified as horizontal or vertical (based on design) and gravity displacement or vacuum type (based on functioning).
- Autoclaves can also be classified as Type “N” and Type “B.”
- Type “N” autoclaves are the ones that do not remove air from the sterilization chamber with the help of a vacuum pump. These are used for solid loads.
- Type “B” autoclaves remove air from the sterilization chamber with the help of a vacuum pump. Wrapped and hollow instruments, which can be sterilized and used later, are to be sterilized by this type of autoclave.
- Importance of bagging the instruments for sterilization.
  - The main aim of bagging or wrapping the instruments prior to sterilization procedure decreases the chances of contamination of the items after the sterilization procedure is complete.
  - Two-layer wrapping of objects should be preferred and the materials used for this can be cotton fabric or muslin, paper, newspaper (Fig. 9.4).

- Monitoring of sterilization.
- Sterilization monitoring is a process by which adequate sterile environment and the effectiveness of the procedure is determined by assessing the biological, mechanical, and chemical parameters.
- The most widely used and accepted parameter of assessing the sterilization procedures is the use of biological indicators (BIs) that directly inhibit the growth of highly resistant microorganisms, rather than merely testing the physical and chemical conditions necessary for sterilization. Since the spores present in a biological indicator are in a much higher number and are highly resistant by nature compared to the other and common microorganisms found on items used for the patient, an inactivated biological indicator indicates that the other microorganisms are killed depicting an effective sterilization procedure. A control BI, from the same lot as the test indicator and not processed through the sterilizer, should be incubated with the test BI; the control BI should yield positive results for bacterial growth.
- Assuring the temperature & the cycle time, and observing the gauges or display for the pressure on the sterilization equipment for each set of items to be sterilized come under the mechanical monitoring of sterilization.
- Certain chemical agents are used to check the efficacy of the sterilization procedure being carried out by undergoing chemical changes in their properties on being exposed to the sterilizing conditions. These chemical agents are called the chemical indicators and these include TST (Time-Steam-Temperature) Strip. This TST strip is to be placed in a big surgical wire basket and when exposed to a critical time, steam, and temperature it undergoes a change in color from yellow to dark blue indicating complete sterilization procedure. Another strip is to be used when double-layered packing of instruments in crepe paper is done to assess the parameters inside the sterilizer.

- Another form of sterilizing monitor is the use of the external indicators. These are usually in the form of Tapes. The main indication of the complete sterilization procedure is the change in color of the strips of the tape from yellow to dark brown or black.
- *How to store the sterilized instruments/objects*
  - There must be a separate area for storing of the sterile instruments and single-use (disposable) products.
  - The storing of the bagged sterilizer objects can be depicted either by the date on which they are sterilized or the procedure for which it is to be used or an event causing it to become contaminated.
  - All the packed items must be carefully inspected before using to verify the integrity of the outer covering and the condition of the packing (dry/wet).
  - Once the packing is assessed and if any breach is seen in the packing, the wrapping should be replaced and the instruments again sent for the sterilization procedure.
  - All the sterilized instruments must be kept in covered drawers.
- Care must be taken that all instruments should be placed away from the place where chances of getting wet are higher.
- *Expiry of the sterilized instruments*
- The expiry of the materials and objects undergoing the sterilization procedure depends on the type and time of the sterilization process, the efficacy of the process, and the handling of the sterilized instruments.
  Once all the sterile conditions are met, 4 weeks is the maximum time for which the items are considered sterilized and safe for use. However, the contaminated instruments are preferred processing and sterilization prior to every procedure.

2. *Ethylene oxide:*
  It is used for sterilization of heat-labile and moisture-sensitive items, supplies, and equipment.
  The operating cycle ranges from 2–24 h and it is a relatively expensive process.
  It can be used for glass, paper surfaces, clothing, plastics and metals, food stuffs, and dental equipment.
  It is unsuitable for fumigating rooms because of its explosive property.
3. *Dry heat:*
  It may be used for sterilization of instruments with cutting surfaces.
  No corrosion occurs with this method of sterilization.
  Dry heat sterilization, which usually occurs consists of hot air oven has typical cycles of 1 h at 171 °C or 2 h at 160 °C.
  Air is a poor conductor of heat and requires a long time for getting the instruments effectively sterilized.
4. *Chemical sterilization and disinfection:*
  This is also a choice for some limited circumstances.
  Some instruments cannot be subjected to high temperatures and in a field environment chemical sterilization may have to be used.
  The disinfectant must remain in contact with the surface for appropriate time.
  Disinfectants include Chlorine solutions, 2–3.2% glutaraldehyde, iodophors, and phenols.

## Operating Room Decorum

1. *Hand hygiene:*

Hand hygiene by operation theater persons is the most efficient way to reduce the risk of spread of infections.

**Fig. 9.5:**
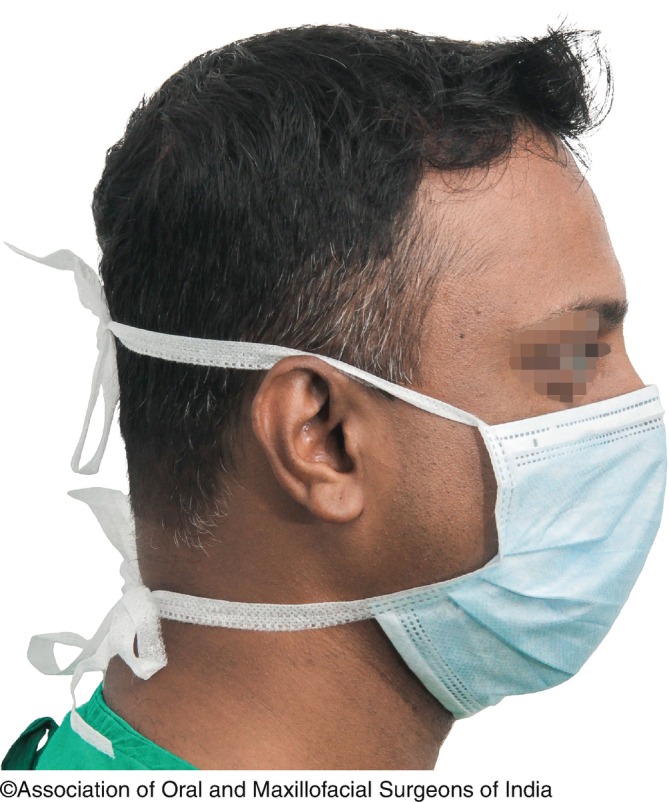
Surgical Mask

**Fig. 9.6:**
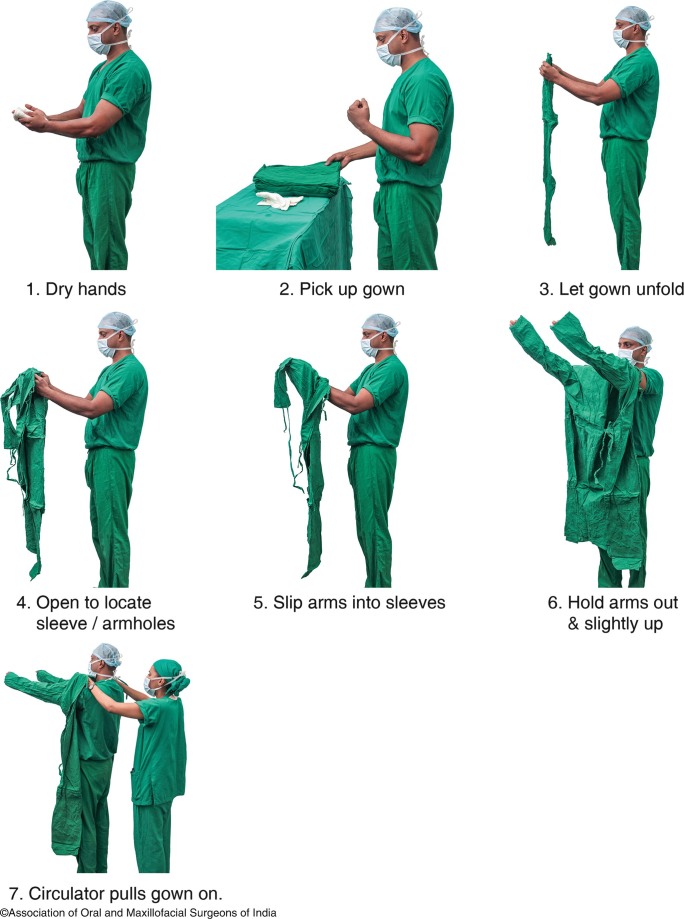
Putting on the sterile gown

**Fig. 9.7:**
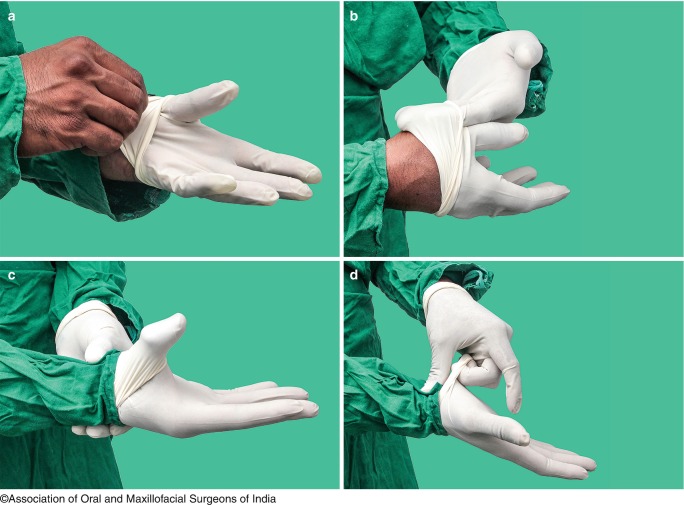
Putting on sterile gloves by open gloving technique

**Fig. 9.8:**
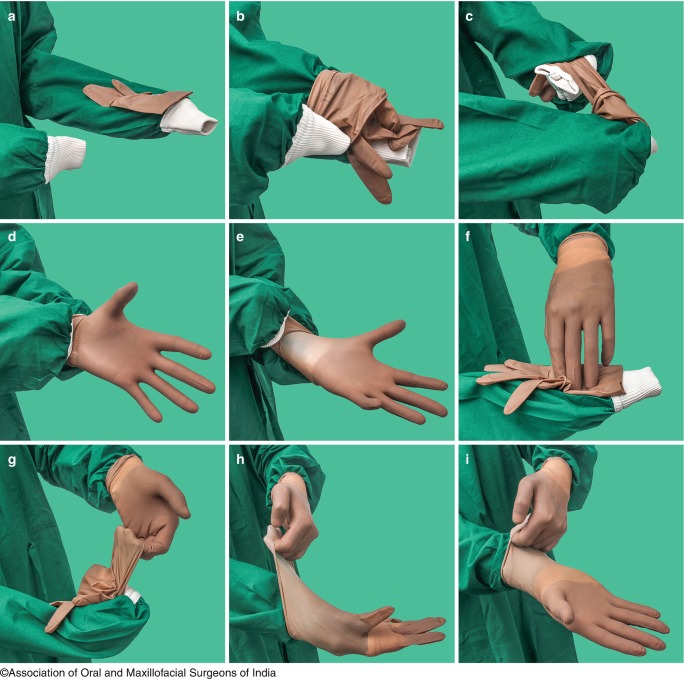
Putting on sterile gloves by closed gloving technique

**Fig. 9.9:**
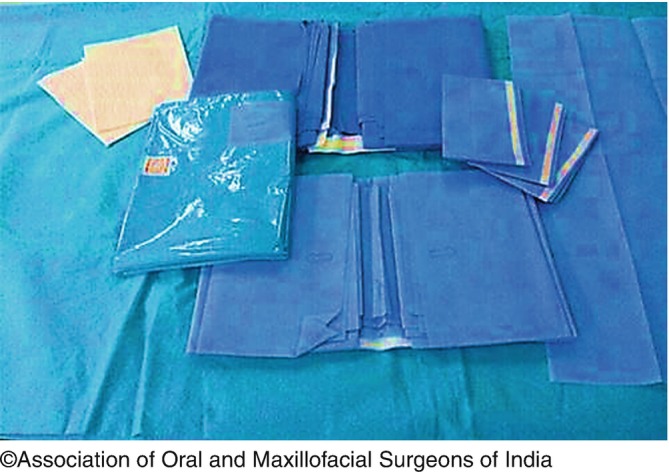
Different types of drapes

2. *Surgical hand wash:*
  Surgical hand wash or surgical hand rub should be carried out prior to the procedure in order to decrease the residing flora of the hand.
  Steps:
    All jewellery from wrists and hands must be removed.
    The temperature of water is to be adjusted so that it is slightly warm. Hands and forearms are to be washed 5 cm above the level of the elbows to remove any particles of dirt.
    Before performing the first scrub of the day, a nail cleaner is used to clean the fingernails and the nail beds.
    The nails should be cut short and no nail polish should be used.
    Antimicrobial agent is to be applied on the hands and in circular motion; lathering should begin at the finger tips of one hand and between the fingers, continuing from the fingertip to 5 cm above the elbow. The same process is to be repeated for other arm and hand.
    The rubbing should be done for a period of 3–5 min.
    Each arm is to be washed separtely at the level of elbow, starting at the fingertips.
      One side of the sterile towel is used to dry the fingertips up to the elbow of one hand and the other side is used to dry the same on the other hand.
3. *Barrier techniques:*
  Barrier techniques are useful where the chances of spread of infection are higher.
  - Head Cover
    Prior or during the procedure, all facial and head hair should be tied properly and covered by means of head covers. Ideally head covers should be disposable and made of soft, nonporous cloth like material. If one has long hair, the hair should be tied in a bun. In situations where tying a bun is not possible, use of helmets or hoods or headgears is of utmost significance.
  - Masks
    Tuberculosis is one of the most common infectious diseases that can spread through airborne route,
    The main aim of using a mask is the prevention of transmission of infectious agents from the member of the operating team to the patient’s wounds and also protecting the operating team members from the splashes and sprays from the patient.
    The masks, which are disposable in nature, are always preferred.
    The mask should be made of synthetic fibers, must be flat with two or three pleats that expand to cover the area up to chin, and should have filters of polypropylene or polyester (Fig. 9.5).

(c) Scrub suits and cover gowns
  These are the pair of garments to be worn over, or instead of regular clothing of the persons involved in the surgical procedures to protect the transmission of any infectious agent present on the regular clothing from the operating personnel to the patient or any other personnel. These should have a simple design, should be comfortable, should be easy to clean and wash, should be economical, easily replicable if damaged and should have minimal place for the contaminants to hide.
(d) Surgical gowns
  These are a loose pair of clothing to be worn over the scrub suits or cover gowns at the time of the surgery to protect both the patient and the operating personnel from transfer of microorganisms, blood or body fluids, and another particulate matter.
  Steps of wearing gown:

- Dry the hands completely and hold the gown in such a manner that it is at the least risk of contamination.
- Slip arms into the sleeves through the armholes, keeping at the shoulder level and away from the body.
- Hold arms out and slightly up.
- The circulating person must pull the gown over the shoulders touching only inside of the gown.
  All the belts and loops are to be tied securely (Fig. 9.6).

(e) Gloves
  They help to protect the operator from infection by bacteria and viruses from patient’s blood. Gloving is essential to protect both the surgeon and the patient from blood-borne viruses and to prevent wound from becoming contaminated with the surgeon’s skin flora.
  The “open gloving” and “closed gloving” technique of donning the gloves should be employed for wearing gloves.

- Steps in wearing gloves by Open Gloving Technique
  - Handwashing must be performed under aseptic conditions.
  - Inspect the outer covering for the integrity. Open the first nonsterile packaging by peeling it completely off the heat seal exposing the inner sterile wrapper, but without touching it.
  - The inner sterile packing is kept on a dry area, without touching the outer surface. Open the package and fold it toward the bottom so as to unfold the paper and keep it open.
  - By using index finger and thumb of one hand, the folded edge of the glove is grasped.
  - In a single movement the other hand is slipped into the glove.
  - The second glove is picked by using the cuff of the other glove and sliding the fingers of the gloved hand into it.
  - In a single movement, slip the second glove on to the ungloved hand while avoiding any contact/resting of the gloved hand on surfaces other than the glove to be donned (Fig. 9.7).

- Steps of wearing gloves by closed gloving technique
  - If the cuffs are not fitting, a tuck is taken in each gown.
  - The circulating person should open the outer covering of the glove and should flip them onto the sterile field.
  - The inner packing containing the gloves is opened carefully and the glove is picked up by the folded cuff edge with the hand covered by the sleeve.
  - The glove is placed on the sleeve of the opposite gown, the palm facing downwards, with the fingers of the glove pointing toward the shoulder.
  - The gloves should be placed in such a manner that the rolled cuff edge of the gloves connects the sleeve to the gown cuff. Bottom cuffed and rolled edge of the glove is grasped at the bottom with the index finger and thumb.
  - With the opposite hand, the outermost edge of the cuff of the gloves is held taking care that the uncovered fingers are not exposed by it.
  - Stretch the glove over the hand
  - By using the opposite hand covered with sleeve, both the cuffs of the sleeve and the glove are seamed and the glove is pulled over the hand.
  - The same procedure is to be followed for the other hand. The fingers are adjusted to properly fit in the glove (Fig. 9.8).

4. The role of drapes:
  Drapes are used during surgical procedure to protect the contacting of the unprepared surfaces/areas and maintaining the sterility of environmental, equipment, and the surrounding of the patients.
  The different drapes available are:-
  Towel drapes, which are used for squaring off the operative site, wrapping syringes & small instruments, and drying of the hands. These must be more resistant to water and must be made of cotton compared to linen.
  Lap sheets are used for covering the patient. They are large and long, usually made of lightweight cotton, and provide limited protection and coverage to the patients or staff or the surface areas.
  Site drapes are made of cotton and have a circular opening in the center that is placed over the prepared operative site. These drapes are primarily intended for use with minor surgical procedures (Fig. 9.9).

## Classification of Surgical Wounds

A widely used classification of surgical wounds is based on an estimate of likelihood of bacterial contamination of the operative site. In 1964 [9], National Academy of Sciences/National Research Council defined five general classes of operations:

1. *Refined-Clean:* Elective operations not drained and primarily closed.
2. *Other-Clean:* Clean cases other than refined clean.
3. *Clean-Contaminated:* Oral cavity, gastrointestinal, or respiratory tract entered without significant spillage, entrance of genitourinary tract in presence of infected urine, entrance of biliary tract in presence of infected bile or minor break in technique.
4. *Contaminated:* Major break in operative techniques (e.g., surgical entrance of unprepared bowel without gross spillage of bowel contents); acute bacterial inflammation without pus; fresh, traumatic wound from a relatively clean source.
5. *Dirty:* Presence of pus or perforated viscous (prior to operation), old traumatic wound, or traumatic wound from a dirty source.

**Table:** 

Currently, this classification has been condensed into four groups for general use, without a subdivision of the clean category and in the modified form it is categorized into four categories [10] 1. *Clean sites (wounds):* • Elective (not urgent or emergency). • Primary closed. • No acute inflammation or transection of tracheobronchial, biliary, gastrointestinal, oropharyngeal tracts. • No technique breaks. 2. *Clean-contaminated sites (wounds):* • Emergency or urgent cases that are otherwise “clean.” • Elective procedures. • Reoperation via “clean incision” within 7 days. • Blunt trauma, intact skin, and negative exploration. 3. *Contaminated sites (wounds):* • Acute nonpurulent inflammation. • Major technique break or major spill from hollow organs. • Entrance of genitourinary or biliary tracts in presence of infected urine or bile, respectively. • Penetrating trauma less than 4 h old. • Chronic open wounds to be grafted or covered. 4. *Dirty sites (wounds):* • Purulence or abscess. • Preoperative perforation of tracheobronchial, biliary, gastrointestinal, oropharyngeal tracts. • Penetrating trauma more than 4 h old.	

## Risk Factors Affecting the Rate of Postoperative Wound Infections

*“Cut Well, Sew Well, Heal Well”* is an axiom favored by surgeons but is not always destined to be true. Altemeir and Culbertson (1965) [11] depicted that the risk of infection varies:

- The risk of infections is proportional directly to the dose of contamination to bacteria.
- The microbial virulence is also directly proportional to the risk of infections.
- The patient’s ability to control and inhibit the resistance is proportional inversely. [12]

**Fig. 9.10:**
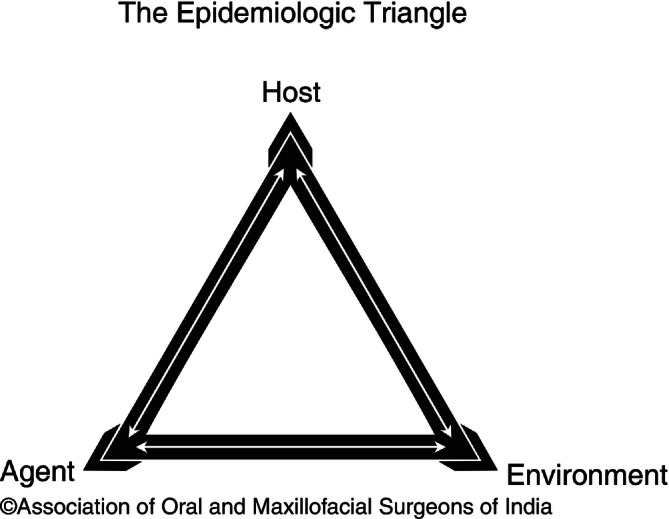
Classical Epidemiological triangle

These factors interact in a complex way to fasten the development of infection. Since the days of Altemeier, clinical and epidemiologic studies have identified the risk factors that affect the rate of postoperative surgical site infection. This can be best explained by the classical epidemiological triangular model, i.e., model of interaction between agent host and environment resulting in disease (Fig. 9.10).

The risk of postoperative wound infection also depends on the patient factors, pre and intraoperative factors (Table 9.5). Haley et al. (1985), in the Study of the Efficacy of Nosocomial Infection Control “(SENIC),“ [13] identified four independent and additive risk factors for postoperative wound infection. These factors are operation on the abdomen, operation lasting for more than 2 h, contaminated or dirty wounds (NRC CLASSIFICATION), and the presence of more than three discharge diagnoses.

**Table 9.5:** Factors that predispose a patient to infection

*Patients factors*	Preoperative factors	Intraoperative factors
*Age* • Malnutrition • Obese persons • Underlying diseases • Hidden infections • Immunosuppressive therapy • Any recent surgery • Longstanding illness	• Longer duration of hospital admission • Improper scrubbing prior to procedure • Removal of hair • Preoperative prophylactic coverage	• Contamination at the time of procedure • Longer duration of the procedure • Any foreign object • Transfusion reactions occurring at the time of the procedure

- Hair removal should not be performed on routine basis to decrease the risk of surgical site infection. Razors should not be used to remove the hair as they increase the chances of infections [14]. Electric clippers with disposable heads must be used only for removing hair few hours prior to the surgical procedure.
- The treatment protocols and the condition of the patient during the hospital stay make them more prone to the spread of infections.
- Wound dressings: Cover surgical incisions with an appropriate interactive dressing at the end of the operation.

### Factors Influencing the Development of Nosocomial Infections

#### The Microbial Agent

Hospital infections occur as a result of a variety of microorganisms. The microorganisms causing the disease may be divided into the following categories:

1. Conventional pathogens are the ones that are capable of causing a disease in normal and healthy person.
2. Conditional pathogens are the ones that initiate the onset of a disease in persons who have decreased immunological response to infection or when implanted directly into sterile area or tissues.
3. Opportunistic pathogens are the ones that normally do not harm its host but can cause disease when the host’s resistance to the microorganisms is low.

During the stay in a hospital, the patient comes in contact with various kinds of microbial agents (such as viruses, fungi, parasites, and bacteria), which are the main cause of the occurrence of infections in a patient. This exposure is not the only reason for the development of hospital-acquired infections. The other possible reasons are the host’s natural defense mechanisms (healthy/compromised/immunosuppressed), conditions present in a hospital environment, microbiology of the microbial agents (characteristic features), infective material present on the microbial agent, resistance to the antimicrobial agents, and other factors. The hospital-based infections may be a result of the spread of infection from one person to the other or by the residential flora of the patient or from any contaminated sources or from sources of the external origin (e.g., airborne diseases).

Following are the factors that are the main sources of spread of hospital-based infections:

#### Susceptibility of the Patient

Patient factors that lead to the occurrence of infection are age, host’s immune response, presence of any disease, and interventions, which either help in diagnosing or treating any condition. Patients, either infant or older individuals, are at a higher risk of acquiring infections. Patients having a compromised immune system, undernourished, having some underlying chronic disease (AIDS, leukemia, malignant tumors, renal failure, diabetes mellitus, etc.), undergoing irradiation therapy, all at the highest risk of being infected by the hospital-based infections. Certain processes like catheterization, biopsy, intubations, etc. make the patient more vulnerable to these infections.

#### Resistance of the Bacteria

Resistance of bacteria to antimicrobial agents is seen with the prolonged or prophylactic use of these agents. The microbial agents present normally in the human flora have both sensitive and resistant strains. Some antimicrobial agents have their action on sensitive strains by suppressing their activity, whereas the resistant strains are still active. These active resistant strains are the major cause of the development of resistance against the antimicrobial agents. Examples of resistant microbial agents are Multiresistant Klebsiella, strains of pneumococci, staphylococci, etc. In low-income or middle-income countries, this is the major problem faced due to unavailability and unaffordability of the better drugs.

#### Month of Operation

The possible explanation for increased infection rates in summer is the relatively high environmental temperature leading to humid climate, resulting in excessive sweating. In addition, excessive sweating results in the displacement of bacteria lodged in skin appendages to the surface.

#### Use of Electric Cautery

The use of electric cautery for cutting and coagulation during surgery causes more inflammation; more necrosis and abscess than the conventional use of scalpel and thus it increases the susceptibility of tissue for infection. Soballe et al [15], in their experimental study, found that electric cautery lowers the contamination threshold for infection of laparotomies and concluded that electric coagulation current should be used only when the need for meticulous hemostasis outweighs the considerably increases risk of infection.

#### Duration of Operation

There is a direct correlation between the infection rate and time taken for the procedure. This may be the result of the more complicated operations being of longer duration, increased wound contamination from airborne bacteria, increased damage to the tissues due to large exposure of the wound, and increased manipulation; moreover, local resistance of tissue is reduced due to drying.

#### Spread of Infection

**Fig. 9.11:**
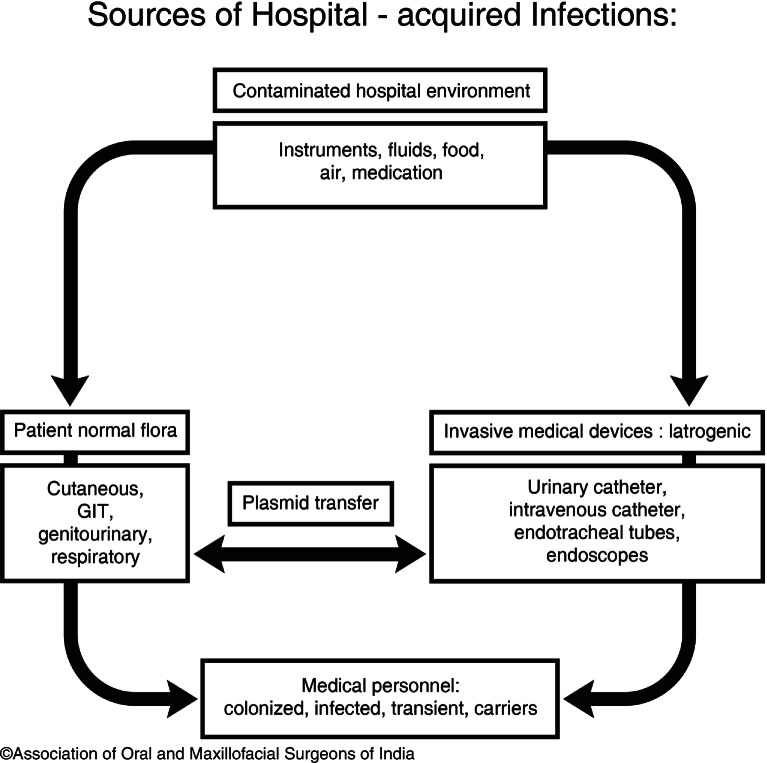
Sources of hospital-acquired infections

The microbial agents are spread by different ways in a health care setup. The different ways with which the agents can spread are contacting, droplets, airborne route, via vehicle, and vector-borne (Fig. 9.11).

Sources of the infectious agents are either external, i.e., from one person to the other, or from the hospital’s environment; or internal, i.e., within the patient himself.

- *Contacting* is the most frequent and important route of spread of infections. It is further of two types, i.e.
  - *Direct* contacting is the spread of infection directly from the person infected from the infection to other normal who has increased chances of being infected. It also occurs among two patients where one acts the source of microbial agents and the other becomes the one to receive the infection.
- *Indirect* contacting is the spread of infection indirectly from one person/object to the other. It involves contact of a susceptible host with a contaminated intermediate object. Droplets spread results due to generation of droplets from the source person mainly during coughing, sneezing, and talking, and during the performance of certain procedures such as bronchoscopy, which can travel a shorter distance via air and get deposited on the body of the other susceptible person.
- *Airborne transmission* results from spreading of small particulate matter (approximately 5 pm or smaller in size) through the air, which remain suspended in the air for a longer period of time. These particles are passed from one individual to the other by the process of inhalation of these particles. The main diseases to be spread via the airborne route are Tuberculosis, Influenza, Common cold, etc.
- *Vehicle spread* is the spread of infectious microbial agents to the individuals by means of contaminated items such as food, water, medications, devices, and equipment.
- *Vector*-borne transmission occurs when vectors such as mosquitoes, flies, rats, and other vermin transmit microorganisms.

## COVID 19 and Maxillofacial Surgery

The occupational risk of acquiring viral diseases has been well known in Maxillofacial Surgery [16]. The WHO announced the Corona virus pandemic also known as COVID-19 as a public health emergency of international concern on January 30, 2020.

Coronaviruses are RNA viruses infecting many species of animals including humans, name coronavirus was derived from corona meaning crown like because of the morphology observed for these viruses in the electron microscope. This family of viruses includes Middle East Respiratory Syndrome (MERS-CoV), Severe Acute Respiratory Syndrome (SARS-CoV), and novel Coronavirus (n CoV).

Coronavirus is enveloped having round, spherical, or sometimes pleomorphic structure, with size ranging from 80 to 120 nm in diameter, containing a positive-strand RNA. The virus is made up of lipid bilayer envelop, membrane protein, and nucleocapsid, these structures also protect the virus when the virus is outside the host cell. The lipid bilayer forms the viral envelop which anchors the membrane protein, envelop protein, and the spike protein. The spike protein (S-protein) is responsible for the crown-like structure of the coronavirus as it forms the protrusions from the surface which bind to the host cells.

### Mode of Transmission of SARs-CoV 2

Human to human transmission is due to respiratory droplet transmission and contact transmission. Spread occurs through coughing, sneezing, spitting, contacting the body fluids of the infected patient. The COVID-19 can remain infectious on inanimate surfaces from 2 h to 9 days, depending on the humidity, temperature, surface type, and viral load [17]. It has become an occupational threat to health care providers worldwide.

Some of the known routes of spread of infection to health care providers are:

- Manual ventilation with a bag and mask
- Intubation
- Open endotracheal suctioning
- Bronchoscopy
- Cardiopulmonary resuscitation
- Sputum induction
- Surgery on the lungs
- Nebulizer therapy
- Noninvasive positive pressure ventilation (BIPAP, CPAP)
- An autopsy on the lungs
- High-speed devices such as those used for surgery, post-mortem
- Dental procedures

Aerosols generating procedures (AGP) create widespread environmental contamination and therefore pose a greater risk of transmission of infection to healthcare workers. Oral and maxillofacial surgeons are particularly vulnerable to this transmissible disease by way of the droplet or aerosol transmission due to the area of work and the type of instrumentation [18].

The incubation period is long and unpredictable ranging from 0 to 27 days with a mean of 6.4 days. Recent studies have shown that asymptomatic patients and those within the incubation period are also potential spreaders of the disease [19].

### Symptoms

Symptoms may range from mild symptoms to severe respiratory distress and some patients may be asymptomatic.

- Common symptoms may include fever, cough, fatigue, shortness of breath, loss of taste, and smell.
- Less common symptoms may include myalgia, headache sore throat, and chills.
- Rare symptoms may include nausea, vomiting, nasal congestion, diarrhea, palpitation, and chest congestion.

### Radiographic Findings

- **Chest X-ray** may show findings that of atypical pneumonia showing bilateral consolidations in lateral lower lobes, bronchovascular thickening.
- **Chest CT** ground-glass opacity and areas of consolidation laterally.

### Testing and Laboratory Findings

The real time polymerase chain reaction (RT-PCR) of respiratory secretions from bronchoalveolar lavage, endotracheal aspirate, and nasopharyngeal or oropharyngeal swab is the definitive test.

Other laboratory findings include lymphopenia, increased prothrombin time, and mildly raised CRP and ESR.

### General Preventive Measures

COVID 19 has shifted the focus on teleconsulting which includes tele screening, telemedicine, and triage. Telemedicine should be practiced whenever possible to decrease the footfall.

Thorough history should be obtained from the patient regarding COVID 19 illness and elective procedures should be postponed and only emergent conditions should be taken up for surgery.

Patient should be called on the basis of appointments, time between two appointments should be sufficient enough to perform all necessary sanitization measures and ensure minimal patient to patient overlap. In the waiting area, posters should be displayed to encourage hand hygiene and the wearing of masks and the area should be well ventilated. A minimum of 2 m distance should be maintained between the individuals.

Extraoral radiographs should be preferred as an alternative to intraoral periapical radiographs.

In the operatory there should be minimum personnel present, Air Conditioners should be avoided, doors and windows are advised to be kept open. The operating room should be spacious with adjoining two rooms for donning and doffing of the PPE’s. No touch sensor-based sanitizer dispenser should be installed at the entry and exit of OR [20].

All the surfaces of equipment like OR table, motor drills etc., should be covered with plastic sheets and sheets to be changed after every patient.

OT’s should be equipped with HEPA filter (0.1 micron efficiency) and high frequency of air changes (ideally 25 per hour) should be ensured to reduce the viral load [20].

High-volume suctions should be used with one-third of suction jars prefilled with povidone-iodine solution.

The Povidone Iodine solution has been shown to have significant viricidal activity up to 3 h and it has been advised to coat the oral cavity and nasal passages of both the patient and the operating team before the surgery [21, 22].

Fogger machines with 0.5% sodium hypochlorite can be used for sterilization of dental chairs, tables, doors, doorknob, etc. [20]. One-minute contact of the chemical ensures viral kill.

### Personal Protective Equipment

- Masks including three-ply surgical masks, N95, N99 (FFP3), and the Powered air-purifying respirators (PAPR’s) provide viral filtration in the increasing order. It is important to mention that N95 masks were not found to be adequate to prevent transmission in Chinese surgeons and PAPR’s had to be used to prevent transmission from COVID patients [23].
- Surgical gloves.
- Goggles and cover all gowns.
- Triple protection gowns and face shield protect the surgeon from exposure to any kind of infected aerosols, body fluids of the patient. Disposable gowns are made of nonwoven material and protect from liquid penetration. Personal protective equipment like basic kit 45 GSM, medium kit 70 GSM for surgery, and advance kit for ICU 180 GSM are recommended to be worn [20].

### Specific Precautions to Be Taken During Surgery

- Surgery on COVID patient should be performed in a negative pressure theater or in airborne infection isolation room [20].
- Focus should be on minimally invasive techniques whenever possible which could reduce the time of surgery and/or reduce aerosol generation.
- Local anesthesia with appropriate sedation should be preferred over GA.
- The surgeon should enter the theater after 20 min following intubation with complete PPE to minimize the exposure to aerosols [23].
- Scalpels should be preferred over cautery.
- Use of high-power drills, oscillating saw, and forceful irrigation should be avoided.
- Absorbable sutures should be used to avoid unnecessary trips of the patient [18].

As the COVID-19 situation is a dynamic evolving one and there is no definitive treatment available proper planning and implementation of infection control protocols are key to preventing transmission of the disease in the Maxillofacial settings.

## Control of Nosocomial Infections

CDC elucidated certain guidelines to check the nosocomial infections in the Study on efficacy of nosocomial infection control (SENIC). These guidelines have decreased the emergence of hospital-acquired infections by a greater number. However, any breach in the infection control procedures can lead to spread of such infections.

Certain conditions leading to improper infection control and spread of such infections are:

1. Improper sterilization and disinfection procedures.
2. Presence of contaminants in the food or water
3. Environment of the health care setup.
4. Untrained and inadequate personnel
5. Insufficient knowledge of Infection control principles and practices among the staff.
6. Antibiotic abuse both in the community and in the hospital.

### Prevention from Nosocomial Infections

Following are the steps that can be taken by the staff in a hospital setup to prevent the spread of such infections:

- Isolation rooms for persons suffering from contagious diseases such as COVID 19 and tuberculosis.
- Hand-scrubbing practices prior to performing any procedure.
- Disinfecting all the areas in a health care setup.
- Adequate sterilization and disinfection protocols to be followed everywhere.
- The wound dressings should be changed at an appropriate time with the use of proper agents.
- The antimicrobial agents should be carefully used. Prolonged use should be avoided whenever possible to avoid the emergence of the resistance.
- Proper management of the biomedical waste.

## Conclusion

Preventing and controlling infections is the key factor in improving care and ensuring safety of both the patient and the health care worker. Infection control addresses factors related to the spread of infections within the operation theater complex (whether patient-to-patient, from patients to staff and from staff to patient, or among staff), including prevention (via hand hygiene/hand washing, cleaning/disinfection/sterilization, vaccination, monitoring).

Integrated infection control in the operation theater has various aspects, ranging from its designing ,environmental cleaning , management of biomedical waste and adherence to theatre attire. Use of Personal Protective Equipment (PPE) including gloves, gowns, face masks ,respirators and full face visors are essential to minimize risks of occupational infections. Whether in developed or developing country, where resources are limited, thorough knowledge about the principles of infection control and a little ingenuity will suffice to solve the problem of hospital-acquired infections.

Surgical site infections are a result of microbial invasion in a sterile atmosphere. The main sources of microbial invasion in the operating theater include the atmosphere of the operating theater, the medical and the paramedical staff present at the time of the procedure, surgical instruments, and the patient at times also. Proper designing of operation theater, appropriate microbiological monitoring, proper sterilization, and strict adherence to barrier techniques form the basis to prevent infections in an operating environment.
